# Associations between serum mitokine levels and outcomes in stable COPD: an observational prospective study

**DOI:** 10.1038/s41598-022-21757-5

**Published:** 2022-10-15

**Authors:** Carlos A. Amado, Paula Martín-Audera, Juan Agüero, Bernardo A. Lavín, Armando R. Guerra, Pedro Muñoz, Ana Berja, Ciro Casanova, Mayte García-Unzueta

**Affiliations:** 1 Department of Pulmonology, Hospital Universitario Marqués de Valdecilla, Av Valdecilla SN, 39005 Santander, Spain; 2 University of Cantabria, Santander, Spain; 3 Department of Clinical Biochemistry, Hospital Universitario Marqués de Valdecilla, Santander, Spain; 4 Servicio Cántabro de Salud, Santander, Spain; 5 IDIVAL (Instituto de Investigación Biomédica de Cantabria), Santander, Spain; 6 Servicio de Neumología-Unidad de Investigación, Hospital Universitario La Candelaria, Universidad de La Laguna, Tenerife, Spain

**Keywords:** Predictive markers, Prognostic markers, Chronic obstructive pulmonary disease

## Abstract

Mitokines (Humanin (HN), GDF15 and FGF21) are produced as a result of mitochondrial dysfunction and may have major roles in chronic inflammation, malnutrition and exercise capacity in people with COPD. Except for GDF15, studies on this subject are lacking. A total of 165 patients with stable COPD and 49 smokers without COPD were enrolled. We assessed their serum mitokine levels and clinical characteristics at baseline. We recorded moderate and severe exacerbation for the next 12 months. Baseline serum HN (p = 0.037) and GDF-15 (p = 0.013) levels were higher in the COPD group. High HN levels were independently associated with a high risk of exacerbation (HRE) (OR 2.798, 95% CI 1.266–6.187, p = 0.011), malnutrition (OR 6.645, 95% CI 1.859–23.749, p = 0.004), and 6MWD (OR 0.995, 95% CI 0.991–0.999, p = 0.008), and future moderate (HR 1.826, 95% CI 1.181–2.822, p = 0.007) and severe exacerbations (HR 3.445, 95% CI 1.357–8.740, p = 0.009). High GDF15 levels were associated with HRE (OR 3.028, 95% CI 1.134–8.083, p = 0.027), 6MWD (OR 0.995, 95% CI 0.990–0.999, p = 0.017) and predicted desaturation in 6MWT (OR 3.999, 95% CI 1.487–10.757, p = 0.006). High FGF21 levels were associated with HRE (OR 2.144, 95% CI 1.000–4.600, p = 0.05), and predicted future severe exacerbation (HR 4.217, 95% CI 1.459–12.193, p = 0.008). The mitokine levels were higher in patients with COPD than smokers without COPD, and were associated with important clinical outcomes such as exercise capacity and COPD exacerbation. Among the mitokines, HN showed the strongest association with COPD and may serve as a future risk biomarker in this disease.

**Trial registation** NCT04449419.

## Introduction

Chronic obstructive pulmonary disease (COPD) is a leading cause of morbidity and mortality worldwide^1^. COPD is a heterogeneous disease in which distinctive characteristics, such as low exercise capacity^2^, low muscle mass^3^ or severe COPD exacerbation^4^, are associated with poorer prognosis. However, clear evidence indicates the presence of impaired mitochondrial structure and function in the lungs, immune cells and skeletal muscles in COPD, possibly because of the chronic effects of excessive reactive oxygen species^5–10^.

Mitochondrial stress elicits the production of various circulating cellular stress response molecules called mitokines, which function as autocrine, paracrine and endocrine signals. Humanin (HN), a peptide encoded in mitochondrial DNA, sends a systemic signal of mitochondrial stress. Circulating HN induces a global cytoprotective effect, because it regulates oxidative phosphorylation, and activates the synthesis of antioxidants and chaperones for unfolded proteins in many tissues^11–17^. Monocytes, alveolar cells and eosinophils express the HN receptors, but the highest expression of these receptors is found in lung macrophages^18^. Human primary mitochondrial diseases (mitochondrial DNA mutations and nuclear DNA mutations in mitochondrial-targeted proteins) and diseases associated with mitochondrial dysfunction are characterized by high circulating HN levels^19–23^. However, no studies to date have examined serum HN levels in COPD.

Stressed mitochondria send signals to the nucleus that activate nuclear genes encoding peptides, such as Growth and Differentiation Factor 15 (GDF15) or Fibroblast growth factor 21 (FGF21), which are also considered mitokines^24^. GDF15 is an inflammation- and metabolism-associated pleiotropic hormone. This mitokine is a well-known marker of morbidity and mortality in COPD and other diseases^25,26^, whereas FGF21 is considered a metabolic hormone and a marker of nutritional stress^27^. As with HN, no studies have described FGF21 in COPD.

We hypothesized that, because of generalized mitochondrial dysfunction, serum HN, GDF15 and FGF21 would be elevated in COPD, and would be associated with outcomes related to muscle mass and function and hence increased risk of exacerbation.

## Methods

This was an observational prospective study performed in a COPD outpatient clinic in a third level hospital in Spain from November 2018 to December 2020. The study protocol is registered at ClinicalTrials.gov (https://clinicaltrials.gov/ct2/show/NCT04449419). The Ethics Committee of our Institution (2018.276) approved the study. All patients provided informed written consent to participate in this study.

### Participants

We recruited patients with COPD during routine visits to the dedicated COPD outpatient clinic. Control individuals were smokers without COPD who were recruited from the smoking cessation clinics from our institution.

The inclusion criteria were as follows: (1) patients with COPD according to the GOLD Guidelines^28^ with age above 40 years, with or without continuous oxygen therapy or (2) age and sex matched smokers without COPD.

The exclusion criteria were as follows: (1) patients with COPD exacerbation 8 weeks prior to inclusion in the study, (2) patients receiving treatment with pulmonary rehabilitation during the study or 6 months before the inclusion period, (3) patients with a previous diagnosis of coronary artery disease, heart failure, patients with respiratory diseases different from COPD, rheumatological diseases or cancer, (4) patients with C-reactive protein levels higher than 2.5 mg/dL or elevated creatine kinase levels and (5) patients with a glomerular filtration rate < 50 mL/min/1.73 m^2^.

### Measurements

We performed spirometry and 6 min walk test (6MWT) according to the Spanish Society of Pulmonology and Thoracic Surgery (SEPAR) protocol^29,30^: patients were asked to walk as far as they can in 6 min in a 30-m straight corridor without any interruption. At the end of the test, the distance walked by the patients and dyspnea were recorded. We estimated body composition with a bioelectrical impedance device (OMROM BF511, Omrom, Japan). We measured maximum hand grip strength with a GRIP-A hand dynamometer (Takei, Niigata, Japan). The diagnosis of disease-associated malnutrition was determined according to the ESPEN consensus (BMI < 18.5 kg/m^2^ or 18.5–22 kg/m^2^, combined with low fat-free mass index (FFMI) (< 17 kg/m^2^ for men and < 15 kg/m^2^ for women)^31^. At the time of entry into the study, patients were categorized as having high risk of exacerbation (HRE) if they had two or more moderate COPD exacerbation events or one severe COPD exacerbation event, according to GOLD^28^, during the previous year. Oxygen desaturation (OD) was defined as a fall in SpO_2_ ≥ 4% or SpO_2_ < 90%^32^. We measured serum creatinine, albumin, uric acid and creatine kinase with Siemens traceable enzymatic method assays (Atellica Analyzer, Siemens, Germany).

Serum HN, GDF15 and FGF21 levels were measured with specific sandwich immunoassays (Human Putative Humanin Peptide MT-RNR2 ELISA, CSB-EL015084HU, Cusabio Biotech, TX, USA; Thermo Fisher Scientific Human GDF-15 ELISA, EHGDF15, CA; and RayBioR Human FGF-21 ELISA, ELH-FGF21, RayBiotech, GA) according to the standard protocols of the mentioned commercial kits.

We obtained early morning blood samples from all participants after they had signed the consent form to participate. Samples and data from patients included in this study were preserved by the Biobank Valdecilla (PT17/0015/0019), integrated in the Spanish Biobank Network, and were processed according to standard operating procedures with the appropriate approval of the ethical and scientific committees.

After entry into the study, patients were followed up for 12 months. We recorded moderate COPD exacerbation (exacerbation in patients treated with antibiotics and/or systemic corticosteroids) and hospitalization due to severe COPD exacerbation prospectively, on the basis of reports by the patients during follow-up visits (6 and 12 months after study entry), and the medical records from the hospital and primary care. Physicians unaffiliated with this study made the diagnosis of exacerbation and the decisions to hospitalize the patients.

### Statistical analysis

Data are presented as mean ± SD for normally distributed data or median (interquartile range) for nonparametric data. We calculated sample size in *Stata Statistical Software: Release 15*. College Station, TX: StataCorp LLC), with an α risk of 0.05 and a β risk of 0.2. Differences between groups were analyzed with unpaired t tests for parametric data or Mann–Whitney tests for nonparametric data. We evaluated for normal distribution with the Kolmogorov–Smirnov test. Evaluation of HN, GDF15 and FGF-21 as a dichotomized variable, with a cut-off at the median, resulted in the best discriminative power for our outcomes, returning the lowest Akaike information criterion value, in concordance with other similar studies^33,34^. We set the cut-off point for 6MWD at 350 m, according to the BODE index^2^. We evaluated cross-sectional associations with univariate and multivariate logistic regression, with high versus low circulating mitokines and 6MWD as the outcome variables. We used Kaplan–Meier survival analysis estimates to calculate the proportion of participants experiencing an event over time. We performed univariate and multivariate analysis with the Cox proportional risk analysis in SPSS Software version 25.00 for PC to identify risk factors associated with moderate COPD exacerbation and severe COPD exacerbation. We considered differences to be significant if the p values were less than 0.05. All reported p values are two-sided.

### Statement of ethics

This study complies with internationally accepted standards for research practice and reporting. The Ethics Committee of our Institution approved the study (2018.276). All patients gave informed written consent to take part in this study.

## Results

### Characteristics of patients and controls

**Figure 1:**
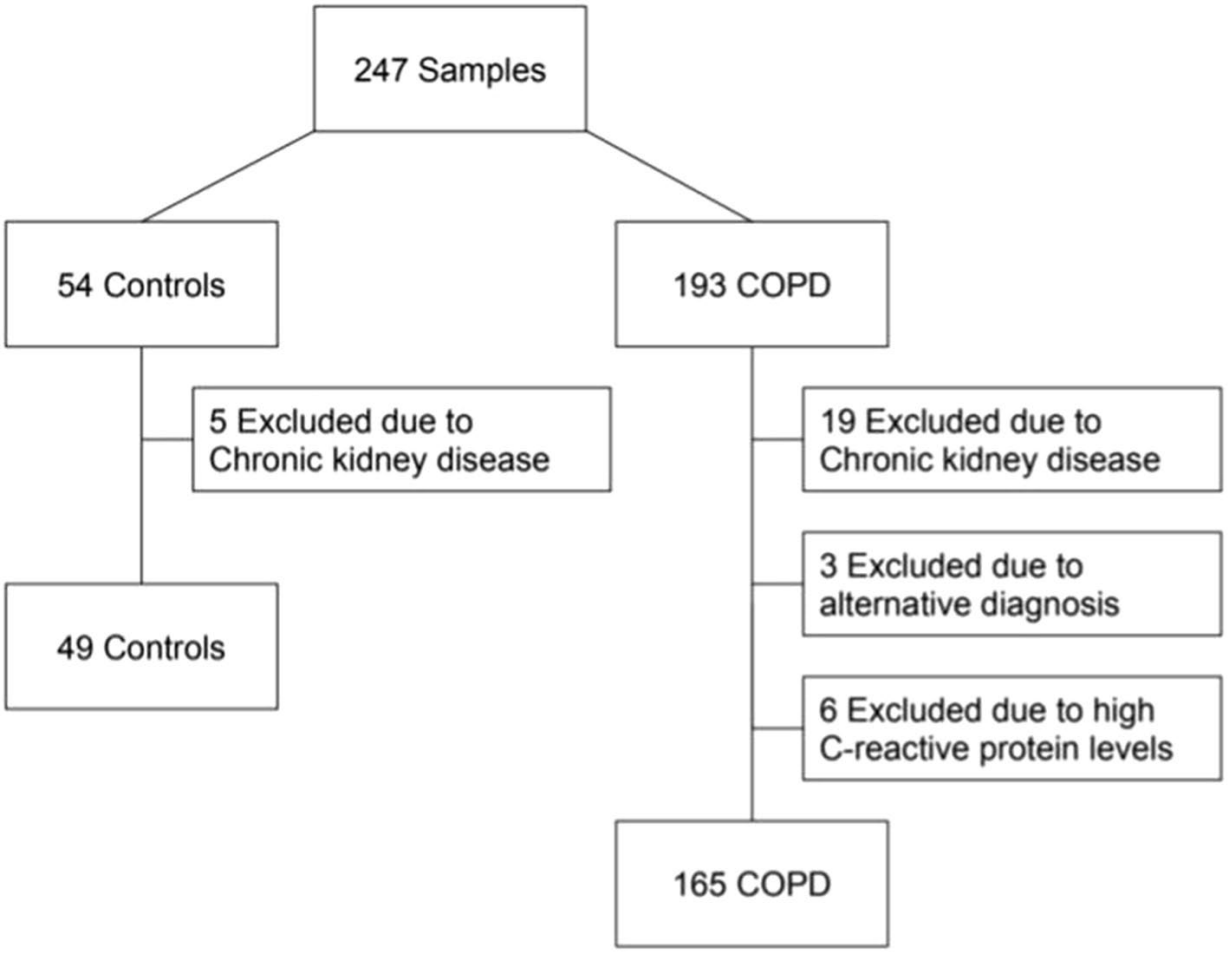
Flowchart for patient selection.

We included 165 patients and 49 sex and age matched controls in the study (Fig. 1). Table 1 shows demographic, clinical and biochemical data. The mean age of the patients was 68 ± 7.4 years, and 65.5% were men. There was a high prevalence of current smokers (29.7%), and most had moderate or severe airway obstruction. The control group had normal lung function, lower CAT and Charlson index values and higher 6 min walk distance (6MWD) than patients with COPD. No patients were treated regularly with systemic steroids. HN and GDF15 levels were higher in the COPD group (256 (60–507) pg/mL and 1244 (913–1716)), respectively, vs. controls (186 (39–338) pg/mL and 1050 (736.5–1487.5) pg/mL). FGF21 levels did not differ between patients and controls. No significant correlations between HN and GDF15, HN and FGF21, or GF15 and FGF21 concentrations were observed in the patients. Notably, we found a positive correlation between GDF-15 and HN (p = 0.015, r = 0.355), and between GDF-15 and FGF-21 (p = 0.049, r = 0.289) in the control group (data not shown).

**Table 1:** Demographic, clinical and biochemical characteristics of controls and COPD patients.

Variable	COPD n = 165	Control group n = 49	p
Age (years)	68 ± 7.6	66.2 ± 6.34	0.116
Sex Male n (%)	108 (65.5%)	29 (59.18%)	0.498
FVC (mL)	**2664 ± 810**	**3435 ± 971**	**< 0.001**
FVC (%)	**83.3 ± 69**	**100 ± 19**	**< 0.001**
FEV1 (mL)	**1250 (870–1725)**	**2610 (2030–2930)**	**< 0.001**
FEV1 (%)	**52 (36.5–68)**	**95 (84–109)**	**< 0.001**
FEV1/FVC	**50 (38.6–60.3)**	**75 (72–78)**	**< 0.001**
Weight (kg)	74.5 ± 16.3	75.92 ± 14	0.579
BMI (kg/m^2^)	27 (24.1–31.6)	27.5 (25.2–29.76)	0.82
6MWD (m)	**427 (330–490)**	**525 (447–572)**	**< 0.001**
Maximum hand grip strength (kg)	30 (23–38)	31 (25–41)	0.409
FFMI (kg/m^2^)	18.8 ± 2.9	18.6 ± 1.5	0.802
CAT score	**13 (7–19.3)**	**3 (1–5.25)**	**< 0.001**
Charlson	**1 (1–2)**	**1 (0–2)**	**0.018**
mMRC score 0/I/II/III/IV	**42 (25.5)/49 (29.7)/45 (27.3)/29 (17.6)**	**39 (79.6)/9 (18.4)/1 (2)/0 (0)/0 (0)**	**< 0.001**
Current smokers n (%)	49 (29.7)	21 (42.9)	0.085
Patients with malnutrition n (%)	**34 (20.6)**	**4 (8.1)**	**0.017**
GOLD 1/2/3/4 n (%)	22 (13.3)/69 (41.8)/51 (30.9)/23 (13.9)	–	–
GOLD A/B/C/D n (%)	52 (31.5)/51 (30.9)/13 (7.9)/49 (29.7)	–	–
High risk of exacerbation n (%)	61 (37)	–	–
1 or more admissions in the previous year n (%)	31 (18.8)	–	–
ICS treatment n (%)	86 (52.1)	–	
ACO n (%)	60 (36.3)		
Diabetes mellitus n (%)	26 (15.7)	8 (16.3)	0.870
Humanin (pg/mL)	**246 (69–507)**	**186 (39–338)**	**0.037**
GDF-15 (pg/mL)	**1244 (913–1716)**	**1050 (736.5–1487.5)**	**0.013**
FGF-21 (pg/mL)	321.9 (176.85–514.05)	242.1 (136.5–396.9)	0.088
Albumin (g/dL)	4.8 ± 0.3	4.78 ± 0.27	0.695
Creatinine (mg/dL)	0.83 (0.69–0.96)	0.82(0.7–0.94)	0.975
Uric acid (mg/dL)	6.2 ± 1.88	5.8 ± 1.49	0.149
CK (UI/L)	66 (44–95)	68 (41–93)	0.910

*FVC* forced vital capacity, *FEV1* forced expiratory volume in the first second, *mMRC* modified Medical Research Council Dyspnea score, *CAT* COPD Assessment Test, *ICS* inhaled corticosteroids, *ACO* asthma COPD overlap, *GOLD* Global initiative for Chronic Obstructive Lung Disease, *BMI* Body Mass Index, *FFMI* Fat Free Mass Index, *6MWD* 6 Minute Walk Test Distance, *CRP* C-reactive protein, Bold font indicates statistical significance.

### Baseline associations of mitokines with COPD disease characteristics

Table 2 shows the associations of mitokines with COPD characteristics at baseline. Univariate logistic regression indicated that high levels of HN were associated with baseline HRE, 6MWD, malnutrition, FFMI and diabetes mellitus. In addition, multivariate logistic regression indicated that high levels of HN were independently associated with baseline High risk of exacerbation (HRE) (2 or more exacerbations during previous year or 1 previous admission) (OR 2.798, 95% CI 1.266–6.187, p = 0.011), malnutrition (OR 6.645, 95% CI 1.859–23.749, p = 0.004) and 6MWD (m) (OR 0.995, 95% CI 0.991–0.999, p = 0.008), whereas FFMI was not.

**Table 2:** Associations between chronic obstructive pulmonary disease characteristics and levels of humanin, GDF-15 and FGF-21 (dependent variables) using uni and multivariate logistic regression.

Variable	High levels of humanin				High levels of GDF-15				High levels of FGF-21			
	Unadjusted		Adjusted		Unadjusted		Adjusted		Unadjusted		Adjusted	
	OR (95% CI)	p	OR (95% CI)	p	OR (95% CI)	p	OR (95% CI)	p	OR (95% CI)	p	OR (95% CI)	p
Age (years)	1.007 (0.967–1.048)	0.745	0.971 (0.917–1.028)	0.313	**1.063 (1.019–1.11)**	**0.005**	1.012 (0.946–1.082)	0.736	1.013 (0.971–1.056)	0.552	0.983 (0.930–1.038)	0.538
**Sex**												
Male	1		1		**1**		1		1		1	
Female	0.851 (0.447–1.619)	0.623	1.619 (0.568–4.615)	0.368	**0.433 (0.224–0.837)**	**0.013**	**0.231 (0.070–0.755)**	**0.015**	1.472 (0.756–2.869)	0.256	0.591 (0.216–1.621)	0.307
**Smoking status**												
Former-smoker	1		1		1		1		1		1	
Current-smoker	1.06 (0.543–2.069)	0.865	0.754 (0.326–1.746)	0.510	0.580 (0.295–1.142)	0.115	1.441 (0.531–3.912)	0.473	1.473 (0.738–2.938)	0.272	1.627 (0.728–3.635)	0.236
**Exacerbation**												
0–1	1		1		1		1		1		1	
** > 1**	**3.255 (1.675–6.326)**	**< 0.001**	**2.945 (1.324–6.553)**	**0.008**	1.032 (0.551–1.935)	0.921	**3.028 (1.134–8.083)**	**0.027**	1.8 (0.934–3.469)	0.079	**2.197 (1.021–4.728)**	**0.044**
**Body composition**												
** Normal**	**1**		1		1		1		1		1	
Obese	0.587 (0.287–1.200)	0.114	0.475 (0.178–1.269)	0.138	1.195 (0.618–2.312)	0.597	0.49 (0.151–1.586)	0.234	0.784 (0.528–2.334)	0.784	0.799 (0.296–2.152)	0.657
** Malnutrition**	**4.015 (1.558–10.349)**	**0.004**	**6.652 (1.845–23.987)**	**0.004**	**2.319 (1.006–5.343)**	**0.048**	3.624 (0.859–15.286)	0.080	0.728 (0.317–1.671	0.454	0.445 (0.143–1.386)	0.163
6MWD (m)	**0.996 (0.993–0.998)**	**0.002**	**0.995 (0.991–0.999)**	**0.009**	**0.995 (0.992–0.998)**	**< 0.001**	**0.995 (0.990–0.999)**	**0.015**	0.999 (0.996–1.002)	0.492	0.997 (0.994–1.001)	0.129
**Charlson**												
1	1				**1**				1		1	
2	1.171 (0.547–2.504)	0.684	1.016 (0.353- 2.925)	0.977	**10.495 (4.236–26.00)**	**< 0.001**	1.243 (0.347–4.456)	0.738	1.622 (0.677–3.883)	0.278	1.486 (0.550–4.013)	0.434
> 2	1.756 (0.794–3.886)	0.165	0.610 (0.193–1.932)	0.401	**10.833 (4.184–28.05)**	**< 0.001**	**12.98 (3.69–45.454)**	**< 0.001**	1.003 (0.457–2.197)	0.995	1.814 (0.621–5.304)	0.276
FEV1 (%)	0.987 (0.972–1.001)	0.072	1.003 (0.974–1.033)	0.844	0.991 (0.977–1.006)	0.225	0.982 (0.950–1.016)	0.296	1.002 (0.987–1.017)	0.836	0.999 (0.971–1.029	0.960
FVC (%)	0.996 (0.982–1.011)	0.604	1.015 (0.986–1.045)	0.322	0.992 (.978–1.007)	0.3	1.015 (0.983–1.047)	0.372	1 (0.985–1.016)	0.972	1.002 (0.975–1.029)	0.877
FFMI (kg/m^2^)	**0.886 (0.793–0.990)**	**0.032**	1.12 (0.897–1.397)	0.318	1.024(0.913–1.149)	0.683	0.956 (0.742–1.232)	0.729	1.038 (0.927–1.162)	0.522	0.926 (0.733–1.157)	0.479
Diabetes mellitus	**0.409 (0.173–0.968)**	**0.042**	2.397 (0.815–7.049)	0.112	**2.937 (1.211–7.123)**	**0.017**	0.702 (0.208–2.372)	0.569	0.580 (0.230–1.46)	0.248	0.515 (0.177–1.499)	0.224

High levels of humanin = humanin higher than median (> 246 pg/mL). High levels of GDF-15 = GDF-15 higher than the median (> 1244 pg/mL). High levels of FGF-21 = FGF-21 higher than the median (> 321.9 pg/mL). Exacerbations = Need for antibiotic or systemic corticosteroids, Malnutrition = BMI < 18.5 kg/m^2^ or between 18.5 and 22 kg/m^2^, combined with low FFMI (< 17 kg/m^2^ for men and < 15 kg/m^2^ for females, high risk of exacerbation = 2 or more exacerbations during previous year or 1 previous admission. *6MWD* 6 Minute Walk Test Distance, *FEV1* forced expiratory volume in the first second, *FVC* forced vital capacity, *FFMI* Fat Free Mass Index, Bold font indicates statistical significance.

At baseline, high levels of GDF15 were associated with age, female sex, malnutrition, 6MWD, greater prevalence of comorbidities and diabetes mellitus. In addition, multivariate logistic regression revealed that high levels of GDF15 were independently associated with female sex (OR 0.235, 95% CI 0.072–0.770, p = 0.017), HRE (OR 3.028, 95% CI 1.134–8.083, p = 0.027), 6MWD (OR 0.995, 95% CI 0.990–0.999, p = 0.017) and greater prevalence of comorbidities (OR 14.92, 95% CI 4.694–47.619, p < 0.001).

At baseline, FGF21 was not associated with any disease characteristics. However, multivariate regression indicated that high levels of FGF21 were associated with HRE (OR 2.144, 95% CI 1.000–4.600, p = 0.05).

### Baseline mitokines as predictors of 6MWT

Forty-eight patients walked less than 350 m in the 6MWD (36 patients with high HN, 33 patients with high GDF15 and 31 patients with high FGF21). Univariate logistic regression indicated that age (p = 0.012), sex (p = 0.006), mMRC dyspnea score (p < 0.001), percentage of FEV1 (p < 001), HRE (p = 0.011), high serum HN (p = 0.01) and high serum GDF15 (p = 0.003) were predictors of low 6MWD. Otherwise, smoking status, the Charlson index value and high FGF21 levels were not predictors of low 6MWD. Multivariate logistic regression analysis indicated that age (OR 1.102, 95% CI 1.027–1.184, p = 0.007), female sex (OR 5.374, 95% CI 1.875–15.401, p = 0.002), the mMRC dyspnea score (OR 3.254, 95% CI 1.748–6.057, p < 0.001) and high levels of HN (OR 3.231, 95% CI 1.212–8.613, p = 0.019) (Table 3) were predictors of low 6MWD. With the same model, neither high GDF15 nor high FGF21 were independent predictors of low 6MWD.

**Table 3:** Logistic regression analysis showing factor associated with walking less than 350 m in 6-min walk test (dependent variable).

Variable	B	p	OR	95% CI OR	
				Lower	Upper
Age (years)	0.098	0.007	1.102	1.027	1.184
Sex (female)	− 1.682	0.002	5.374	1.875	15.401
Charlson index	0.284	0.145	1.329	0.906	1.949
mMRC dyspnea score	1.180	< 0.001	3.254	1.748	6.057
FEV1 (%)	− 0.024	0.093	0.976	0.949	1.004
High risk of exacerbation	0.377	0.479	1.458	0.513	4.142
Current smoker	− 0.537	0.327	0.585	0.200	1.709
High humanin levels	1.173	0.019	3.231	1.212	8.613
K	− 8.339	0.001	< 0.001		

High humanin levels = humanin higher than median. High risk of exacerbation = 2 or more exacerbations during previous year or 1 previous admission. FEV1 = forced expiratory volume in the first second.

A total of 62 patients presented OD (39 patients with high HN, 42 patients with high GDF15 and 38 patients with high FGF21). Univariate logistic regression indicated that the mMRC dyspnea score (p < 0.001) FEV1 (%) (p < 0.001), HRE (p < 0.001), high serum HN (p = 0.006) and high serum GDF15 (p = 0.001) were predictors of OD. Otherwise, High FGF21 levels were not predictors of OD. Multivariate logistic regression analysis indicated that the mMRC dyspnea score (OR 1.764, 95% CI 1.08–2.88, p = 0.023), FEV1 (%) (OR 0.957, 95% CI 0.933–0.982, p = 0.001), high levels of HN (OR 2.551, 95% CI 1.077–6.040, p = 0.033) and high levels of GDF15 (OR 3.999, 95% CI 1.487–10.757, p = 0.006) (Table 4) were independent predictors of low OD.

**Table 4:** Logistic regression analysis showing predictors of oxygen desaturation in 6-min walk test (dependent variable).

Variable	B	Wald	p	OR	95% CI OR	
					Lower	Upper
Age (years)	0.010	0.095	0.758	1.010	0.950	1.073
Sex (female)	0.106	0.051	0.821	1.112	0.444	2.783
Charlson index	− 0.141	0.636	0.425	0.868	0.614	1.229
mMRC dyspnea score	0.567	5.148	0.023	1.764	1.080	2.880
FEV1 (%)	− 0.044	10.912	0.001	0.957	0.933	0.982
High risk of exacerbation	− 0.567	1.546	0.214	0.567	0.232	1.386
Current smoker	− 0.401	0.645	0.422	0.670	0.252	1.781
High GDF-15levels	1.386	7.539	0.006	3.999	1.487	10.757
High humanin levels	0.936	4.533	0.033	2.551	1.077	6.040
High FGF-21 levels	− 0.551	1.479	0.224	0.577	0.237	1.401
K	0.175	0.007	0.932	1.192		

High GDF-15 levels = GDF-15 higher than the median (> 1244 pg/mL). High humanin levels = humanin higher than median (> 246 pg/mL). High FGF-21 levels = FGF-21 higher than the median (> 321.9 pg/mL). High risk of exacerbation = 2 or more exacerbations during previous year or 1 previous admission. FEV1 = forced expiratory volume in the first second. Oxygen desaturation (OD) was defined as ≥ 4% reduction between pretest and posttest arterial oxygen saturation (Δ SpO_2_ ≥ 4%) and posttest SpO_2_ < 90% measured by pulse oximetry.

### Baseline mitokines as predictors of moderate exacerbation

During the 12-month follow-up period, 93 of the 169 patients presented moderate COPD exacerbation. (57 in the high HN group, 44 in the high GDF-15 group and 35 in the high FGF21 group).

**Figure 2:**
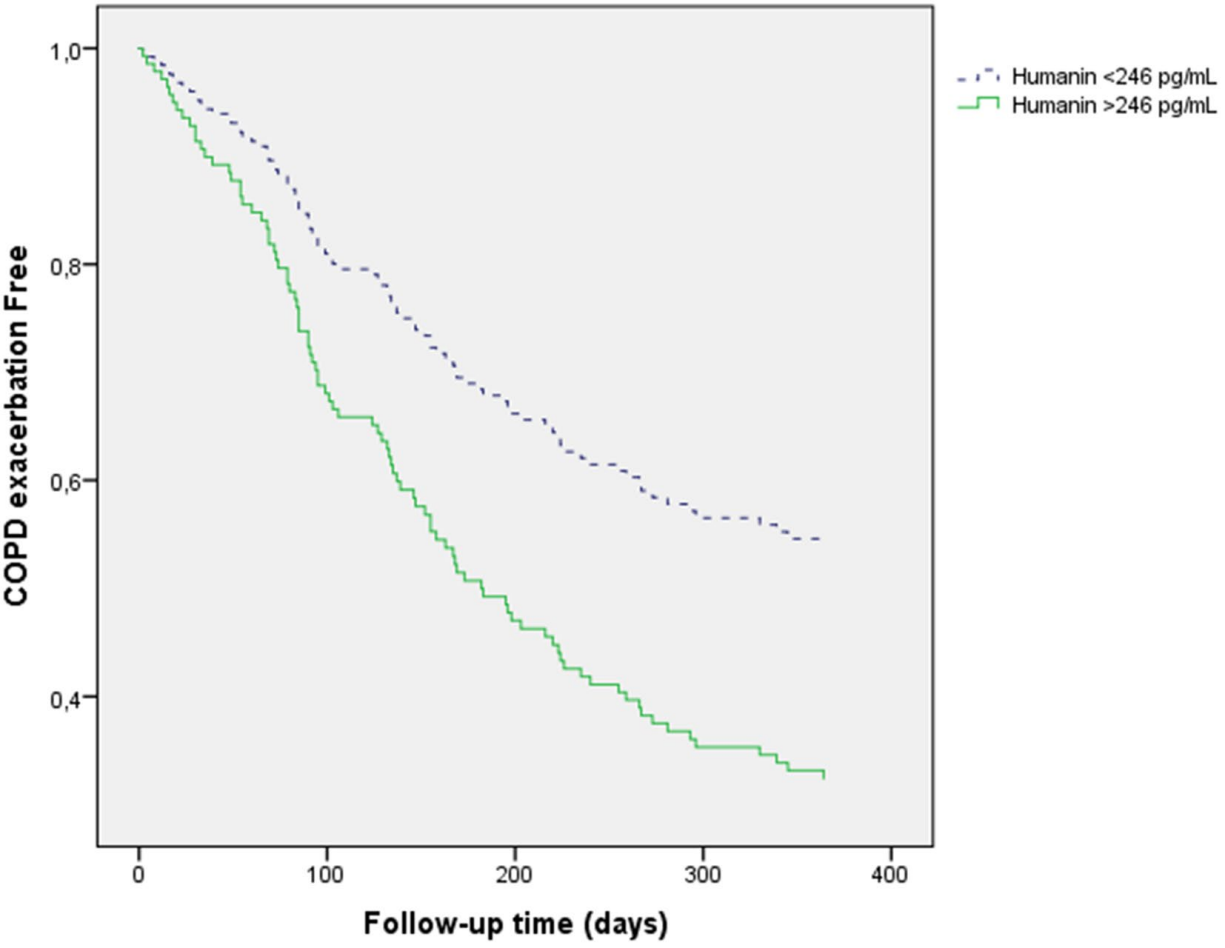
Serum HN levels as predictors of moderate COPD exacerbations.

Univariate Cox proportional risk analysis indicated that high HN (p = 0.001), the mMRC dyspnea score (p = 0.037) and HRE (p = 0.001) were risk factors for COPD exacerbation, whereas high GDF15 or FGF21 levels were not. Multivariate Cox proportional risk analysis revealed that HRE (HR 1.842, 95% CI 1.158–2.928, p = 0.01) and high HN (HR 1.826, 95% CI 1.181–2.822, p = 0.007) were independent risk factors for moderate COPD exacerbation (Fig. 2; Table 5). With the same model, high GDF15 (HR 1.386, 95% CI 0.869–2.209, p = 0.171) and FGF21 (HR 0.986, 95% CI 0.640–1.518, p = 0.948) were not independent risk factors for moderate COPD exacerbation (data not shown).

**Table 5:** Multivariate Cox proportional risk analysis showing predictors of moderate COPD exacerbations (dependent variable).

Variable	B	p	HR	95% CI HR	
				Lower	Upper
Age (years)	0.029	0.054	1.029	1.000	1.059
Sex (female)	− 0.260	0.257	0.771	0.492	1.209
Current smoker	− 0.258	0.294	0.772	0.477	1.251
mMRC dyspnea score	0.088	0.494	1.092	0.849	1.403
Charlson index	− 0.060	0.475	0.942	0.799	1.110
FEV1 (%)	< 0.001	0.979	1.000	0.988	1.013
High risk of exacerbation	0.611	0.010	1.842	1.158	2.928
High HN levels	0.602	0.007	1.826	1.181	2.822

High humanin levels = humanin higher than the median (> 246 pg/mL). High risk of exacerbation = 2 or more exacerbations during previous year or 1 previous admission. FEV1 = forced expiratory volume in the first second.

### Baseline mitokines as predictors of severe exacerbation

Twenty-nine patients were hospitalized (23 in the high HN group, 20 in the high GDF15 group and 23 in the high FGF21 group).

**Figure 3:**
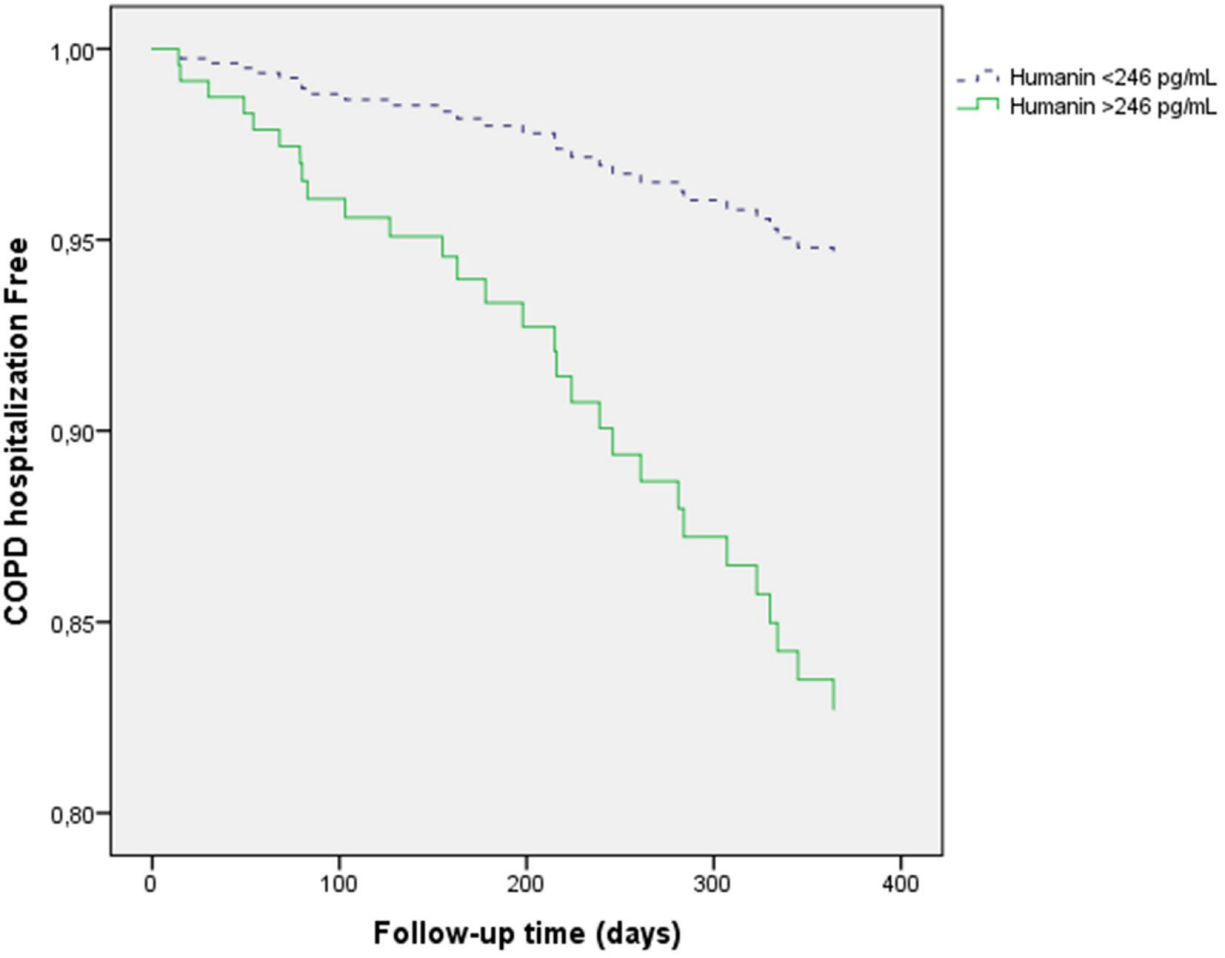
High serum humanin levels (higher than the median) as predictors of severe COPD exacerbations.

Univariate Cox proportional risk analysis indicated that high HN (p = 0.001), the mMRC dyspnea score (p < 0.001), the Charlson index (p < 0.001), FEV1 (p = 0.001) and previous admission for COPD exacerbation (p < 0.001) were risk factors for hospitalization, whereas high GDF15 levels were not. High FGF21 was not associated with the risk of COPD hospitalization (p = 0.079). Multivariate Cox proportional risk analysis indicated that age (HR 1.065, 95% CI 1.007–1.127, p = 0.027), previous admission (HR 2.759, 95% CI 1.198–6.354, p = 0.017), the Charlson index (HR 1.213, 95% CI 1.010–1.465, p = 0.039) and high HN (HR 3.445, 95% CI 1.357–8.740, p = 0.009) were independent risk factors for hospital admission (Fig. 3; Table 6). With the same model, high FGF21 (HR 4.217, 95% CI 1.459–12.193, p = 0.008) was also an independent risk factor for hospital admission (Supplemental file), whereas high GDF15 (HR 1.224, 95% CI 0.493–3.041, p = 0.663) was not (data not shown).

**Table 6:** Multivariate Cox proportional risk analysis showing predictors of COPD hospitalizations (dependent variable).

Variable	B	p	HR	95% CI HR	
				Lower	Upper
Age (years)	0.063	0.027	1.065	1.007	1.127
Sex (female)	− 0.040	0.925	0.961	0.416	2.216
Current smoker	− 0.716	0.103	0.489	0.207	1.155
mMRC dyspnea score	0.475	0.059	1.608	0.983	2.630
Charlson index	0.193	0.039	1.213	1.010	1.456
FEV1 (%)	− 0.016	0.249	0.984	0.958	1.011
Previous admission	1.015	0.017	2.759	1.198	6.354
High HN levels	1.237	0.009	3.445	1.357	8.740

High humanin levels = humanin higher than the median (> 246 pg/mL). Previous admission = 1 or more admission during previous year. FEV1 = forced expiratory volume in the first second.

## Discussion

Our study revealed several novel and important findings: First, patients with stable COPD, compared with smokers without COPD, had significantly higher serum levels of HN and GDF-15, and showed a trend toward elevated FGF21 levels. Second, the levels of these mitokines did not correlate with each other in COPD, thus suggesting that their regulation and/or metabolism are different. Finally, the mitokines were associated with outcomes in patients with COPD, although a different relationship was observed for each mitokine.

The differences among mitokines were consistent with the known metabolic characteristics of each molecule. Whereas the HN gene is under the direct control of mitochondrial DNA (and is indicative of mitochondrial stress when elevated), nuclear DNA encodes the GDF15 and FGF21 genes, and they are only partially under the control of mitochondria through mitochondrial-to-nuclear signaling (ATF3/4, etc.)^35^. In fact, many other stimuli—such as inflammatory cytokines, hypoxia, PPAR-alpha ligands, carcinogens, diet, exercise, lipids and amino acids—modulate GDF15 and FGF21 through specific transcription factors^36–38^.

HN had not previously been studied in the context of COPD. According to our data, HN is associated with malnutrition and 6MWD. Both characteristics indicate that skeletal muscle—a tissue affected by COPD that accounts for half the body weight and is rich in highly active mitochondria—is the most important source of excessive circulating HN, although immune, airway, parenchymal lung cells and pulmonary vasculature cells may also contribute. The increased levels of HN in COPD appear to be a compensatory reaction to protect mitochondria, and hence cells, against generalized oxidative stress. High HN levels indicate not only lower 6MWD but also oxygen desaturation, thus suggesting a relationship with exercise capacity as well as ventilation/perfusion mismatch during exercise. Furthermore, mitochondrial dysfunction associated with COPD-induced low-grade inflammation may also be responsible for the increased HN levels, thus making HN an interesting prognostic biomarker that provides information on malnutrition, skeletal muscle dysfunction and chronic inflammation. In fact, our data show that high circulating HN levels are a prognostic factor for moderate and severe exacerbation in the next year.

Previous studies have shown that GDF15 is a promising, albeit unspecific, biomarker in COPD^33^. GDF15 levels were initially described to be highly elevated in septic patients in critical care and have also been described to be elevated in patients with COPD^39–42^. Our data confirmed these findings. Various explanations have been suggested regarding the factors associated with high GDF15 levels in COPD. Wu et al. and Verhamme et al.^43,44^ have demonstrated that cigarette smoke induces GDF15 in human tracheobronchial epithelial cells. Mutlu et al.^40^ have suggested that generalized inflammation is a factor, because GDF15 levels correlate with levels of CRP, a well-known systemic inflammatory marker. Moreover, GDF15 levels have been found to be lower in stable patients than in patients with exacerbation in at least two studies^39,40^. In another study, higher GDF15 levels have been associated with higher coronary artery scores in patients with COPD, whereas no correlation has been found with common markers of COPD severity^45^. The authors have proposed that high GDF15 may be mediated by asymptomatic atherosclerosis, another cause of chronic low-grade inflammation. Our data also showed that comorbidities were associated with high levels of GDF15, thus suggesting that GDF15 may be elevated in many diseases. In contrast, Patel et al. have shown that in COPD, circulating GDF15 is inversely correlated with exercise capacity^42^, but not with BMI or FFMI. Regarding the prognostic value of GDF15, the longitudinal Bergen COPD study^33^ has importantly found that high concentrations of GDF15 at the time of entry into the study were associated with a higher annual exacerbation rate, mortality, and a faster decline in lung function over 3 years of follow up. In our study, the number of patients was smaller, the follow-up period was shorter, and patients with important comorbidities (heart disease, kidney failure, etc.) were excluded; therefore, our negative results might have been due to less statistical power. Nonetheless, our data suggested that high HN levels may be more specific prognostic factors than high GDF15 levels. Clearly, more studies are needed to clarify these aspects.

FGF21 had not previously been studied in COPD. FGF21 levels were higher in patients with HRE, and were predictive of hospitalization, but the strength of the association was weak, probably because of the many factors that regulate FGF21 levels, some of which are not fully known. Nonetheless, the relationship between FGF21 levels and COPD outcomes is less clear than that observed with HN herein.

Our study has several limitations. Because this was a single center study, these results should be replicated in larger multicenter studies, which should examine other sociodemographic characteristics and diseases known to alter mitokine levels. Further studies are required to demonstrate or exclude a potential role of HN, FGF21 and other “new” mitokines not studied herein^46^, and to evaluate how the serum mitokine measurements change during time. We used stringent criteria to exclude patients with altered pulmonary function and other conditions known to increase mitokine levels (active exacerbation, sepsis, severe inflammation, renal insufficiency, clinical coronary artery disease, etc.); therefore, the results are not generalizable to all patients with COPD, but they add new pathophysiological information. States of low-grade inflammation and clinical asymptomatic atherosclerosis or other asymptomatic diseases were not excluded, although we do not believe that these conditions could have strongly influenced our results. However, our study reveals only associations but not causality.

The main strength of our study is that it was prospective and was specifically designed to evaluate the possible utility of measuring mitokines in a group of well characterized COPD patients differing in obstruction severity and clinical characteristics.

## Conclusion

In conclusion, with a blood mitokine panel, we evaluated GDF-15 and, for the first time, HN and FGF-21 in patients with COPD. The mitokine levels were higher in COPD than in smokers without COPD, and were associated with important clinical outcomes such as exercise capacity and exacerbation. Among the mitokines, HN showed the strongest prognostic value and may serve as a future risk biomarker in this disease. Further studies are needed to confirm our findings.

## Supplementary Information

Supplementary Information.

## Data Availability

The datasets used and/or analysed during the current study available from the corresponding author on reasonable request.

## Abbreviations

COPD: Chronic obstructive pulmonary disease
HN: Humanin
GDF15: Growth and differentiation factor 15
FGF21: Fibroblast growth factor 21
6MWD: 6 Minute walk distance
6MWT: 6 Minute walk test
OD: Oxygen desaturation
SEPAR: Spanish Society of Pulmonology and Thoracic Surgery
BMI: Body mass index
FFMI: Fat free mass index
HRE: High risk of exacerbation
