# Supplementary material for: Associations between serum mitokine levels and outcomes in stable COPD: an observational prospective study
Source: Sci Rep. 2022 Oct 15;12:17315. doi: 10.1038/s41598-022-21757-5 (PMC9569360; doi:10.1038/s41598-022-21757-5)
Supplement: Supplementary file 1 — Supplementary Information. [file 41598_2022_21757_MOESM1_ESM.docx]

**Supplemental file**

**Table:** High serum FGF21 levels (higher than the median) as predictors of severe COPD exacerbations.

|  | | | | | |
| --- | --- | --- | --- | --- | --- |
|  | B | p | HR | 95,0% CI | |
|  |  |  |  | Inferior | Superior |
| Age (years) | 0.037 | 0.213 | 1.038 | 0.979 | 1.100 |
| Sex (female) | 0.275 | 0.511 | 1.316 | 0.580 | 2.988 |
| Current smoker | -0.702 | 0.105 | 0.496 | 0.212 | 1.157 |
| mMRC Dyspnea score | 0.369 | 0.147 | 1.446 | 0.878 | 2.379 |
| Charlson index | 0.295 | 0.003 | 1.343 | 1.105 | 1.633 |
| FEV1 (%) | -0.021 | 0.151 | 0.980 | 0.953 | 1.008 |
| Previous admission | 1.415 | 0.001 | 4.115 | 1.842 | 9.193 |
| High FGF21 levels | 1.439 | 0.008 | 4.217 | 1.459 | 12.193 |

**Figure:** High serum FGF21 levels (higher than the median) as predictors of severe COPD exacerbations.
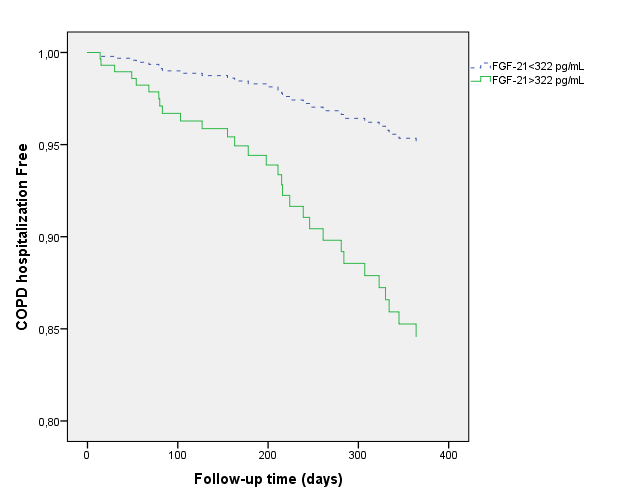


|  |
| --- |
